# The Effect of 12 Weeks of β-Hydroxy-β-Methyl-Butyrate Supplementation after Liver Transplantation: A Pilot Randomized Controlled Study

**DOI:** 10.3390/nu11092259

**Published:** 2019-09-19

**Authors:** Barbara Lattanzi, Michela Giusto, Carlina Albanese, Gianluca Mennini, Daria D’Ambrosio, Alessio Farcomeni, Stefano Ginanni Corradini, Massimo Rossi, Manuela Merli

**Affiliations:** 1 Department of Translational and Precision Medicine, Sapienza University of Rome, 00185 Rome, Italy; Lattanzi.b@gmail.com (B.L.); daria.damb@gmail.com (D.D.); stefano.corradini@uniroma1.it (S.G.C.); 2 Department of Radiological Sciences, Oncology and Pathology, Sapienza University of Rome, 00185 Rome, Italy; carlina.albanese@uniroma1.it; 3 Department of General Surgery “Paride Stefanini”, Liver Transplantation Unit, Sapienza University of Rome, 00185 Rome, Italy; gianluca.mennini@uniroma1.it (G.M.); massimo.rossi@uniroma1.it (M.R.); 4 Department of Public Health and Infectious Diseases, Sapienza University of Rome, 00185 Rome, Italy; afarcome@gmail.com

**Keywords:** β-hydroxy-β-methyl-butyrate, sarcopenia, liver transplantation, appendix skeletal muscle index, dual energy X-ray absorptiometry

## Abstract

Sarcopenia is a frequent complication in liver transplant (LT) recipients. β-hydroxy-β-methyl-butyrate (HMB) has the potential to increase muscle-performance and tropism. Our study aims at evaluating the effect on muscle mass and functioning, and the safety of 12 weeks of HMB supplementation in patients after LT. This is a pilot, randomized study. Male patients undergoing LT were randomly assigned to the HMB or control group. A diet interview, anthropometry and body composition by dual energy X-ray absorptiometry (DEXA) were performed at enrollment (T0), after 12 weeks (T1) and after 12 months (T12). Twenty-two liver transplant male patients were enrolled in the study: 12 in the HMB group and 10 as the control group. At enrollment, demographic, clinical and nutritional data were similar. According to the appendicular skeletal muscle index, sarcopenia was present in 50% of patients. The appendix skeletal muscle mass index (ASMI) showed a significant increase at T1 and T12 in HMB patients, but not in controls. The mid-arm muscle-circumference and hand grip strength also increased at T1 and T12 versus T0 only in the HMB group. No side effects were reported in either group. The study showed a positive effect of HMB in the recovery of muscle mass and strength after LT. HMB supplement in patients after LT was safe and well tolerated.

## 1. Introduction

Malnutrition is an important burden in patients affected by liver cirrhosis with an incidence ranging from 20% to 90%, depending on the population studied and the diagnostic tools used for the diagnosis [1,2,3]. The main component of malnutrition in liver cirrhosis is represented by sarcopenia. The term sarcopenia was first developed to describe a condition of muscle atrophy associated with aging process (“primary sarcopenia”). More recently, a progressive and generalized loss of muscle mass and strength has been recognized in other clinical settings, such as in advanced liver failure, independently of ageing (“secondary” sarcopenia) [4]. In the present paper, the term “sarcopenia” refers to “secondary sarcopenia,” if not otherwise specified.

Sarcopenia is associated with an increased risk of mortality, higher prevalence of portal hypertension-related complications and infections, and longer hospital stays for cirrhotic patients [5,6,7,8].

Sarcopenia represents a negative prognostic factor for survival in patients undergoing liver transplantation (LT) [9]. Furthermore, sarcopenia is associated with prolonged stay in the intensive care unit, longer hospitalization and a higher rate of infections after LT [10,11].

By restoring liver function, LT is expected to ameliorate the patient’s nutritional status. In fact, many metabolic alterations that are involved in causing malnutrition in cirrhotic patients depend on the liver’s inability to regulate energy metabolism and to maintain an adequate protein synthesis. However, unlike other complications of cirrhosis, sarcopenia may not improve and sometimes even worsens after LT [12,13,14,15,16].

The time windows utilized for planning a nutritional intervention in liver transplant patients are represented by the pre-transplant period, taking advantage of the time between entry into the transplant list and LT; the peri-operative period, during the hospital stay; or the post-transplant period, planning nutritional support to be implemented at home after hospital discharge. Randomized trials investigating the effectiveness of pre or post-transplant nutritional support are still lacking, and available data are not encouraging [17]. A few studies reported that a nutritional support, enteral or parenteral, adopted in the first 24–48 h post-transplant, is a valid and effective approach for reducing hospitalization and postoperative complications [18,19,20,21]. On the other hand, one study analyzed the long-term effects of pre and peri-operative immunonutrition support, but failed to demonstrate any improvement in the recovery of lean body mass in the supplementation group after LT [22]. At present, nutritional intervention studies for the treatment of sarcopenia following liver transplantation are not available.

After LT, prolonged bedrest, a hypoactive lifestyle, steroid therapy and the use of anti-rejection drugs, such as inhibitors of mTOR (Sirolimus and Everolimus), may affect recovery of muscle mass and even worsen sarcopenia. In this phase, a nutritional intervention to improve muscle metabolism would be of potential benefit to restore muscle mass, regain muscle function and improve nutritional status. Beta-hydroxy-beta-methylbutyrate (HMB), an active metabolite of leucine, has been identified as a possible nutritional supplement with an anabolic effect, and is able to inhibit muscle proteolysis [23].

**Figure 1:**
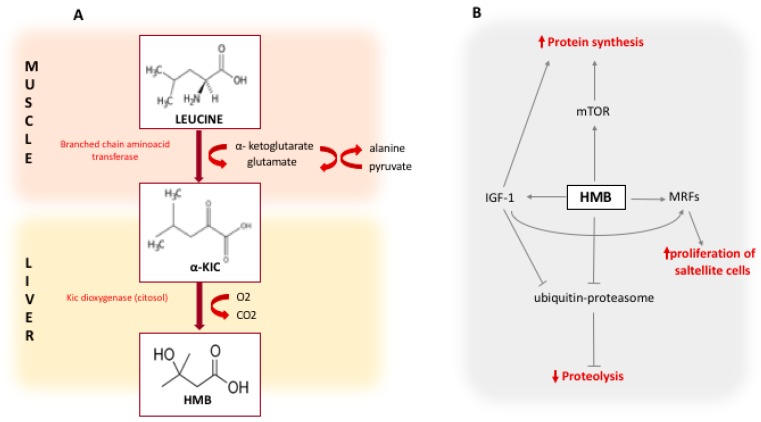
Panel A shows the pathway of HMB synthesis from Leucine. The first reaction is the reversible transamination of leucine to α-KIC by BCAA-aminotransferase. This reaction primarily occurs in skeletal muscle. The second reaction, that occurs primarily in the liver, is the is the metabolization of α-KIC by the cytosolic enzyme α-KIC-dioxygenase to produce HMB; panel B shows the mechanisms of action of HMB. HMB increases the protein synthesis via mTOR and by stimulating the production of IGF-1, and reduces the proteolysis through the inhibition of the ubiquitine-proteasome system. HMB also increases the proliferation of satellite cells by the stimulation of MRFs; HMB: Beta-hydroxy-beta-methylbutyrate; α-KIC: alpha-keto-isocaproic acid; BCAA: branched chain amino acids; mTOR: mammalian target of rapamycin; MRFs: Myogenic regulatory factors; IGF-1: insulin-like growth factor 1.

The potential role of HMB on muscle mass metabolism has been displayed in Figure 1.

The association of anabolic properties targeting mTOR and anti-proteolytic properties, makes supplementation with HMB of potential efficacy for the treatment of sarcopenia after liver transplantation. 

To our knowledge, no studies are available on the effect of HMB supplementation in liver transplanted patients. We, therefore, conducted a preliminary prospective clinical trial aimed at analyzing the effect of HMB in this clinical setting. The primary endpoint of this study was to evaluate the efficacy of 12 weeks of HMB supplementation after a LT in terms of improving muscle mass.

Secondary endpoints were: (1) the safety profile; (2) the long-term effects (6 and 12 months); (3) the effect of HMB supplementation on muscle function.

## 2. Materials and Methods

The study is a single center, preliminarily randomized controlled trial for HMB supplementation versus a control after LT. As the planned sample size was small, to avoid the confounding effect of gender, we decided to include only male patients. All subjects gave their informed consent for inclusion before they participated in the study. The study was conducted in accordance with the Declaration of Helsinki, and the protocol was approved by the Ethics Committee of 23 July 2015 (Cod 3706).

After institutional review board approval, male patients older than 18 years of age undergoing elective LT for end-stage liver disease at the transplant center of the University Hospital Policlinico Umberto I, were enrolled. Patients from other regions, unavailable for serial controls at the outpatient’s clinic, patients with low compliance or those who refused to participate, were excluded.

Patients were enrolled 30 days after LT (T0) when they were out of hospital and clinically stable. Randomization was performed according to a previous computer calculated numerical series. Patients in the HMB group received 3 g/day of HMB, in two doses of 1.5 g dissolved in equal amounts (200 mL) of fruit juice taken twice a day, for 12 weeks; patients in the control group were asked to consume fruit juice (200 mL) twice a day as nutritional supplement.

Dietary advice was given to both groups in order to provide a calorie intake of 25–30 kcal/kg/day and a protein intake of 1.2 g/kg/day. An everyday active lifestyle was encouraged.

Both groups were evaluated at the same time points: enrolment (T0), end of treatment (T1), 6 months (T6) and 12 months (T12) from the inclusion in the study. The study was registered on trial.gov (NCT03234920). At each visit, clinical complications, comorbidities and hospital re-admissions were recorded. Complete biochemical data were registered, and nutritional assessments was performed. Intolerances and/or side-effects for HMB were evaluated during the study and systematically reported.

Nutritional assessment included both the evaluation of muscle mass and function. Muscle mass was evaluated through anthropometry and dual energy X-ray absorptiometry (DEXA), as previously reported [24]. Anthropometry included mid-arm muscle-circumference (MAMC) and tricep skinfold-thickness (TSF) of the non-dominant arm measured using a Harpenden Skinfold Caliper (John Bull British Indicators Ltd., St. Albans, UK).

DEXA was performed to obtain three indices: the appendix skeletal muscle mass index (ASMI), the fat free mass index (FFMI) and the fat mass index (FMI).

Sarcopenia was diagnosed by an ASMI or FFMI <5th percentile versus a control value obtained in a healthy subject matched for age and sex [25].

Muscle function evaluation included the 6-min walk test (6MWT), timed up and go test (TUG) and hand grip strength (HG). The 6MWT measures the distance an individual is able to walk over a total of six minutes on a hard, flat surface. The goal is for the individual to walk as far as possible, being allowed to self-pace and rest as needed. The TUG measures, in seconds, were the time taken by an individual to stand up from a standard arm chair, walk a distance of 3 m, turn, walk back to the chair, and sit down. The HG evaluates, the maximum strength obtained by the hand squeezing a handgrip dynamometer. The subject holds the dynamometer in the hand, with the arm at right angles and the elbow by the side of the body. The maximum isometric effort, is maintained for about 3–5 s. The mean value of 3 measurements with 30 s rest is considered.

## 3. Statistical Analysis

The planned sample size was arbitrarily established to 22 patients, considering the lack of previous data on HMB supplementation in liver transplanted patients and it being a pilot randomized controlled study. Clinical, laboratory and instrumental data were gathered in a pre-established database. For the categorical variables, the Person-Chi-square test or the Fischer exact test were used. For continuous variables the Mann–Whitney Test was used. The analysis of paired data was made for repeated variables. Data were expressed as means and standard deviations, medians and ranges, or percentages when indicated. Values of *p* < 0.05 were considered statistically significant.

## 4. Results

### 4.1. Study Population and Baseline Characteristic

From March 2015 to September 2017, forty-one patients underwent LT at the Transplants Unit of the University Hospital Policlinico Umberto 1, “Sapienza” University of Rome.

**Figure 2:**
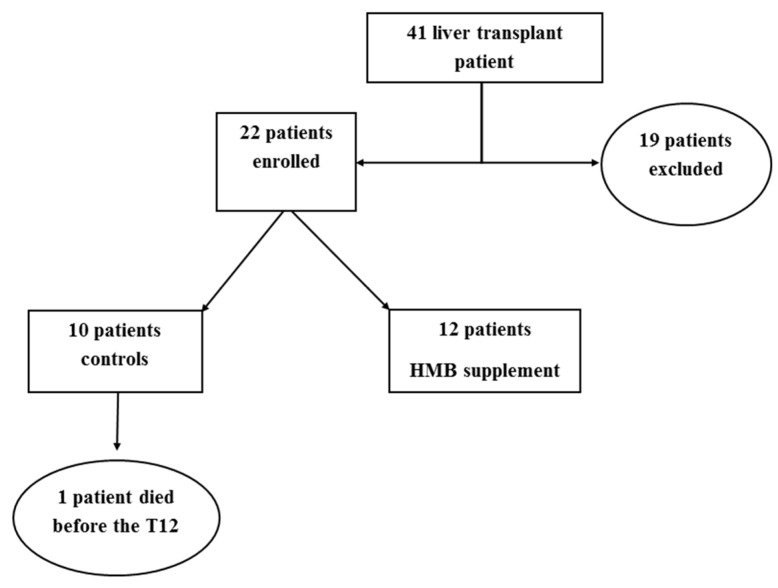
Flow chart of the study.

Twenty-two patients were enrolled in the HMB protocol: 12 in HMB group and 10 in the control group. Nineteen patients were excluded from the study: 13 females, one pediatric patient, three patients who moved out of the region, and two patients who refused to take part to the study (Figure 2).

The patients’ mean age was 59.9 ± 6.2 years. The mean of the model of end stage liver disease (MELD) at transplantation was 16.2 ± 7.5.

The main cause of end-stage liver disease leading to LT was viral cirrhosis (12 patients, 50%), followed by non-alcoholic steatohepatitis (five patients, 23%) and alcoholic cirrhosis (five patients, 23%). A concomitant diagnosis of hepatocellular carcinoma within the Milan criteria was present in 72% of patients at transplantation. None of these patients had a diagnosis of recidivism during the study.

At the time of enrollment (T0), the HMB group and the control group were similar (Table 1).

During follow-up, one patient in the control group died from sepsis at the 2nd month after LT. Due to the short follow up, we decided to remove that patient from further analysis. Thus, the reported results refer to the 21 patients who achieved T12 (nine patients in the control group and 12 patients in the HMB group).

### 4.2. Safety and Tolerability

Compliance in the treatment arm was good and none of the patients reported side effects due to HMB assumption. Therapeutic drug monitoring of immunosuppressive agents in the group of patients receiving HMB supplementation demonstrated stable blood concentrations.

### 4.3. Modifications of Muscle Mass Evaluated by DEXA

Five patients in the control group (50%) and seven patients (58%) in the HMB group were sarcopenic at enrollment (T0).

ASMI showed a significant increase at T1 in HMB patients (6.8 ± 0.7 kg/m² versus 7.4 ± 0.8 kg/ m²; *p* = 0.0003), but not in controls (7.17 ± 1.4 kg/m² versus 7.4 ± 1.17 kg/m²; *p* = 0.4) (Table 2). At the end of the supplementation period (T1), the five patients of the control group were still sarcopenic while in the HMB group, three patients improved (ASMI >5th percentile).

**Figure 3:**
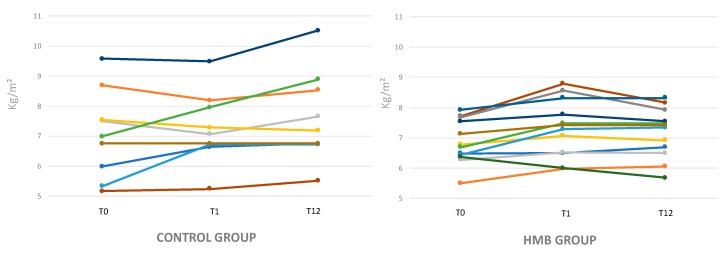
Appendix skeletal muscle index (ASMI) modification in the control and HMB group during follow-up.

At the end of the observation period (T12) the increase in ASMI was confirmed only in the HMB group (ASMI 6.8 ± 0.7 kg/m² versus 7.2 ± 0.7 kg/m²; *p* = 0.006 for HMB group and 7.17 ± 1.4 kg/m² versus 6.84 ± 2.87 kg/m²; *p* = 0.3 control group) (Figure 3). Four patients in the control group and 4 in the HMB group were sarcopenic (ASMI <5°percentile) at T12.

The evaluation of the FFMI showed only minor non-significant changes. FMI showed a trend of increase in both groups, reaching a significance at T12 only in patient of control group (7.1 ± 2 kg/m² versus 8.0 ± 3.2 kg/m²; *p* = 0.02).

### 4.4. Modification of Muscle Mass Evaluated by Anthropometry

In the HMB group, an amelioration of MAMC in comparison to the baseline was observed at T1 (26.1 ± 2.3 cm versus 27.0 ± 2.6 cm; *p* = 0.04) and confirmed at T12 (26.1 ± 2.3 cm versus 27.7 ± 3.6 cm; *p* = 0.03). In the control group, a slight amelioration of MAMC was observed, not reaching statistical significance though (*p* = 0.2).

### 4.5. Muscle Strength and Physical Performance

**Figure 4:**
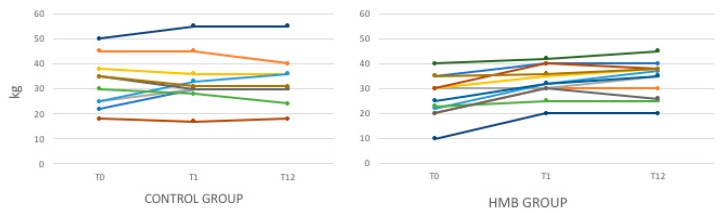
Hand grip strength (HG) modification in the control and HMB group during follow up.

Muscle strength, assessed by HG test, increased significantly in the HMB group, both at T1 (26.6 ± 8.3 versus 32.7 ± 6.4, *p* = 0.0002) and at T12 (33.7 ± 7.6, *p* = 0.001). In the control group, the muscle strength evaluated by HG was substantially stable (Table 2) (Figure 4).

Physical performance, assessed by 6MWT and TUG (time up and go) tests did not show significant changes in both groups.

## 5. Discussion

Sarcopenia is a frequent complication in liver transplant recipients that may persist or even appear de novo [13,14,15,26].

Our pilot study shows that: (1) 12 weeks HMB supplementation improves the skeletal muscle mass recovery in patients after LT and its effect persists at 12 months; (2) HMB is safe and well tolerated in patients after LT; (3) HMB supplementation improves muscle strength up to 12 months after LT.

HMB has been utilized to improve muscle performance in various clinical conditions.

At least three different pathways were detected in which HMB intervenes to modulate the loss of skeletal muscle mass, with studies conducted both in vivo and in vitro [27]. The first anabolic mechanism is the proliferation of myoblasts by the activation of the GH/IGF-1 axis that activates the proliferation, differentiation and survival of myoblasts, conferring atrophy reduction and the improvement of strength [28,29,30]. A role of HMB in the suppression of proteolysis was also demonstrated through inhibition of the ubiquitin-proteasome pathway in models of the neoplastic cachexia [31] and the effect of attenuating muscle atrophy secondary to steroid therapy [32]. Another pathway in which HMB intervenes is that regulated by the enzyme mTOR, a protein kinase responsive to mechanical, hormonal and nutritional stimuli, with a central role in the control of cell growth, by controlling the efficiency of the mRNA translation [27]. All these mechanisms could have been activated in the HMB group.

In a recent systematic review and meta-analysis, Bear and coworkers examined 15 randomized controlled trials involving 2137 patients [33]. The patients included older people in care-home residences or hospitalized; critically ill subjects; patients submitted to orthopedic interventions; and patients affected by gastric, pulmonary or rheumatologic diseases. All experienced muscle wasting as part of their clinical condition and were treated with HMB 3 g/day alone, or in combination with arginine and glutamine, or with various oral nutritional supplements. The majority of the studies adopted a duration of intervention varying between 4 and 24 weeks. In spite of a rather small effect size, differences in the methods for muscle assessment and being cautious about the presence of consistent biases in many of the included studies, the authors concluded with a positive effect of HMB supplementation on skeletal muscle mass and strength in a variety of clinical groups [33].

Our pilot study includes a new category of patients among those showing a beneficial effect of HMB supplementation. Following liver transplantation, hypomobility, chronic immunosuppressive therapy and surgical complications may all be confounding factors contributing to compromising the recovery of skeletal muscle mass. Complications may require re-admission, and this may further slow down the recovery of skeletal muscle mass. In the first period, steroid therapy may worsen pre-existing diabetes and de novo diabetes may also occur, with obvious consequences on muscle anabolism. In our study, the complications requiring re-admission during the observation period (T0-T12) were equally distributed in the two groups. The prevalence of diabetes was also similar, being that a diagnosis of diabetes was present in 57% of individuals in the control group and in 66% (*p* = NS (Not significant)) in the HMB group.

Following our protocol, nutritional counselling was given to each patient, in which we recommended an adequate calorie and a protein intake (25–30 kcals/kg/day ± 10% and 1–1.2 g/kg/day for proteins). From the analysis of food questionnaires, all patients improved their dietary intake during the study. Care was also given to encourage a more active lifestyle, although a specific physical activity was not formally suggested. These recommendations were given also to the control group, but the changes reported were minor and muscle mass and strength failed to improve at 12 weeks and 12 months after LT. On the other hand, the daily consumption of 3 g HMB for 12 weeks was able to induce a statically significant amelioration in muscle mass (ASMI and MAMC) and strength (HG test) during the follow-up.

With regard to fat mass (FMI, evaluated by DEXA) we reported a trend toward increase in both groups, although statistical significance was reached only in the control group. In a previous study, we observed a similar increase in fat mass in liver transplant patients at 6 and 12 months [15]. In some cases, this increment can even lead to overweight and liver steatosis. Although this was not among the aims of our study, it is interesting to note that the increase in fat mass was lower in the group receiving HMB supplementation.

Concerning tolerability and safety, none of the patients discontinued HMB treatment due to side effects and the therapeutic drug monitoring of immunosuppressive agents demonstrated no relevant modifications of the drugs’ blood concentrations. Transaminases, blood urea nitrogen, creatinine, cholesterol, triglycerides and glucose blood levels did not show significant changes during treatment (data not shown). The safety of HMB, in this small pilot study, encourages the possibility of adopting HMB supplementation in liver transplant patients.

Our study recognizes some limitations. First, this is a pilot study with a limited number of patients; second, the compliance of the patients could not be directly measured but was only derived from patients’ reports at clinical visits; third, we adopted a dose and timing of HMB supplementation that could have been further optimized, but our study was not planned to provide information about this. Lastly, due to the limited number of patients, to avoid the confounding effect of gender, we planned to exclude female patients, which could also be considered a bias of the study.

In conclusion, the study demonstrates the tolerability and safety of HMB in patients after liver transplantation and reports a positive effect of this supplementation on the recovery of muscle mass and strength after LT. More studies with larger populations are needed to confirm these results.

## Figures and Tables

**Table 1:** Characteristics of patients at baselines in the control and HMB groups.

Variables	Control Group (*n* = 10)	HMB Group (*n* = 12)	*p*-Value
Age (Years)	59.3 ± 7.3	60.4 ± 5.4	0.68
MELD score at transplantation	16.8 ± 8.4	15.7 ± 7.1	0.70
Hepato Cellular Carcinoma n (%)	8 (80)	8 (67)	0.48
Hospitalization after LT (days)	10 ± 20.4	12 ± 18.25	0.57
Aetiology n (%)			
HCV	4 (40)	6 (50)	
Alcohol	2 (20)	3 (25)	
Autoimmune	1 (10)	0	
Non Alcoholic Steato Hepatitis	3 (30)	2 (17)	0.61
HBV	0	1 (8)	
BMI (kg/m^2^)	25.7 ± 4.9	24.5 ± 3.0	0.48
MAC (cm)	29.8 ± 5.0	28.9 ± 2.8	0.61
TSF (mm)	8.75 ± 2.5	9.0 ± 2.9	0.81
MAMC (cm)	27.1 ± 4.5	26.1 ± 2.3	0.51
FMI (kg/m²)	6.9 ± 4.4	7.1 ± 2.1	0.9
FFMI (kg/m²)	17.5 ± 2.4	16.6 ± 1.5	0.29
ASMI (kg/m²)	7.2 ± 1.4	6.9 ± 0.7	0.53
ASMI [<5° percentile] n (%)	4 (40)	7 (58)	0.39
FFMI [<5° percentile] n (%)	4 (40)	8 (67)	0.21
FMI [>95° percentile] n (%)	3 (30)	1 (8)	0.19
HG (kg)	32 ± 10.2	26.7 ± 8.3	0.17
6MWT (m)	316.5 ± 203.5	334.8 ± 129.2	0.80
TUG (s)	8.2 ± 4.9	9.7 ± 4.5	0.47
Calorie Intake (kcal/kg/die)	23.8 ± 6.3	22.7 ± 8.6	0.76
Protein intake (g/kg/die)	0.7 ± 0.2	0.9 ± 0.2	0.55
Diabetes pre-LT n (%)	5 (50)	6 (50)	0.99
Arterial Hypertension pre-LT n (%)	4 (40)	7 (58)	0.53
Diabetes post-LT n (%)	2 (20)	5 (42)	0.35
Arterial Hypertension post-LT n (%)	5 (50)	7 (58)	0.53

MELD: model of end stage liver disease; BMI: body mass index; MAC: mid-arm circumference; TSF: tricep skinfold-thickness; MAMC: mid-arm muscle-circumference; FMI: fat mass index; FFMI: fat-free mass index; ASMI: appendicular skeletal muscle index; HG: hand grip; 6MWT: 6 minute walk test; TUG: time up and go.

**Table 2:** Comparison of nutritional and performance variables in the control and HMB groups during follow up.

	Control Group (*n* = 9)			HMB Group (*n* = 12)		
Variable	T0	T1	T12	T0	T1	T12
FFMI (kg/m^2^)	17.5 ± 2.4	17.6 ± 2.1	17.9 ± 2.7	16.6 ± 1.4	17.0 ± 1.6	16.8 ± 1.2
ASMI (kg/m^2^)	7.17 ± 1.4	7.4 ± 1.1	6.8 ± 2.87	6.8 ± 0.7	**7.4 ± 0.8** *	**7.2 ± 0.7** *
MAMC (cm)	27.3 ± 5.1	28.7 ± 5.1	28.7 ±4.6	26.1 ± 2.3	**27.0 ± 2.6** ˠ	**27.7 ± 3.6** ˠ
FMI (kg/m^2^)	7.3 ± 4.4	7.6 ± 5.0	**8.7 ± 4.7** ˠ	7.1 ± 2.1	7.7 ± 2.5	8.0 ± 3.2
6MWT (m)	316.5 ± 203.5	312.8 ± 186.0	431.1 ± 125.3	334.8 ± 129.2	389.2 ± 143.8	413.8 ± 151.1
HG (kg)	33.2 ± 10.8	33.5 ± 10.3	33.7 ± 11.1	26.6 ± 8.3	**32.7 ± 6.4** *	**33.7 ± 7.6** *
TUG (s)	8.2 ± 4.9	10.7 ± 2.4	9.6 ± 2.1	9.7 ± 4.5	8.7 ± 2.6	9.1 ± 3.0

Values refer to patients who completed the study for up to 12 months. Continuous data are expressed as means and standard deviations and were analyzed with the Student’s *t* test. The data in bold correspond to statistically significant variations. * *p* < 0.001 compared to T0; ˠ *p* < 0.05 compared to T0; FFMI: fat-free mass index; ASMI: appendicular skeletal muscle mass; MAMC: mid-arm muscle-circumference; FMI: fat mass index; 6MWT: 6 minute walk test; HG: hand grip; TUG: time up and go.

## Abbreviations

β-hydroxy-β-methyl-butyrate	HMB
liver transplantation	LT
dual energy X-ray absorptiometry	DEXA
Appendicular skeletal muscle index	ASMI
Mid Arm Muscle Circumference	MAMC
Triceps Skinfold-Thickness	TSF
fat free mass index	FFMI
fat mass index	FMI
6-min walk test	6MWT
Timed Up and Go Test	TUG
Hand grip strength	HG
branched chain amino acids	BCAA
